# The Digital Competences of Exercise Therapists in Obesity Care: A Step Towards Digital Sovereignty Assessed with the DigCompThExO Questionnaire

**DOI:** 10.3390/healthcare14081037

**Published:** 2026-04-14

**Authors:** Sabine Pawellek, Isabell Estorff, Hagen Wulff, Thomas Wendeborn

**Affiliations:** 1 Department of Sport Pedagogy, Faculty of Sport Science, Leipzig University, 04109 Leipzig, Germany; hagen.wulff@uni-leipzig.de (H.W.); thomas.wendeborn@uni-leipzig.de (T.W.); 2 Institute of Exercise and Public Health, Faculty of Sport Science, Leipzig University, 04109 Leipzig, Germany; isabell.estorff@uni-leipzig.de

**Keywords:** obesity, exercise therapy, digital competence, digital sovereignty, digital health, professional education

## Abstract

Background/Objectives: Digital obesity therapy requires exercise therapists with adequate digital competences, yet training opportunities remain limited. This study provides the first application of the DigCompThExO questionnaire to assess exercise therapists’ digital competences and their predictors in obesity therapy, addressing digital sovereignty as an educational outcome and informing future training programs. Methods: A cross-sectional online survey assessed self-perceived digital competences among German-speaking exercise therapists in obesity care using the validated DigCompThExO questionnaire (14 items). Descriptive and regression analyses examined personal (age, gender, qualification) and contextual (type of therapy, therapeutic targets) predictors of overall digital competence, with correction for multiple testing. Results: Of 203 therapists (mean age 33.3 ± 5.9 years), ‘Teaching Strategies’ yielded the highest scores, ‘Selection Criteria’ the lowest. Regression analysis (n = 202) accounted for a substantial proportion of variance in overall digital competence (R^2^ = 0.801, adjusted R^2^ = 0.790, *p* < 0.001), with the digitally pursued therapeutic target body awareness emerging as significant predictor (B = 0.18, p_FDR = 0.003). Conclusions: This study provides initial insights into the digital competence profiles of exercise therapists in obesity therapy. In exploratory analysis, the therapeutic target of digitally fostering body awareness was the only predictor that remained significant after correction. The findings suggest that targeted education in data protection, media reflection, and the communication of exercise-related therapeutic targets may be relevant to support digital competence development.

## 1. Introduction

Obesity, defined as a chronic, relapsing, multifactorial disease characterized by excessive adiposity that impairs health, represents one of the most pressing public health challenges of the 21st century [1]. Effective obesity management therefore requires multimodal therapeutic approaches addressing behavioral, nutritional, medical and exercise-related components [2]. In Germany, for example, obesity treatment is typically embedded in structured rehabilitation and aftercare programs within the public healthcare and social insurance system. However, current obesity therapy remains limited due to insufficient individualization and persistent infrastructural barriers [3]. Digitally delivered therapy offers an alternative, providing location-independent access and flexible adaptation to patients, thereby supporting sustainable weight reduction [4,5].

In Germany, digital obesity treatment is increasingly supported by digital health applications, which are gradually integrated into clinical practice by complementing on-site treatment with app-based or remote components. Nevertheless, implementation remains limited, despite initial studies outlining methods and content for digital obesity therapy [6]. This likely reflects multiple factors across stakeholder perspectives. Structural barriers include limited digital infrastructure, funding mechanisms, and evidence on effectiveness relative to time and resources. Patient-related factors such as feasibility in everyday life, adaptability to individual needs, and sustained engagement may further influence uptake. From therapists’ perspective, concerns about effectiveness and increased workload remain relevant, as digital tools introduce additional pedagogical and organizational demands requiring both technical skills and reflective decision-making [7]. Among these perspectives, therapists represent a key leverage point, as they are directly involved in the implementation and delivery of digital interventions. Accordingly, addressing these demands requires a broader educational orientation, conceptualized in this study as digital sovereignty, which denotes reflective, self-determined, and responsible professional engagement with digital technologies beyond mere technical proficiency [8,9]. Digital sovereignty is not directly measurable but is approached indirectly through the assessment of digital competences as its foundational prerequisites. Therefore, digital competence and digital sovereignty are conceptually distinct: while digital competency encompasses the knowledge, skills, and attitudes required to use digital technologies, digital sovereignty focuses more on functional and person-centered professional education. Accordingly, digital sovereignty can be understood as an overarching outcome of digital competence development. From this perspective, the systematic identification and assessment of relevant digital competences, as well as their targeted development based on existing competence profiles, represent key steps in supporting the integration of digital approaches in obesity therapy.

To meet this requirement, structured competence frameworks and corresponding assessment instruments are essential to operationalize and evaluate digital competences in professional practice. Educational research has addressed this need through frameworks such as Digital Competence framework for Educators (DigCompEdu), which conceptualizes digital competences across six domains [10], demonstrating the value of competence-based assessment for identifying deficits and informing targeted training programs [11].

However, effective training requires the consideration of determinants shaping competence levels. Prior research indicates that digital competences are associated with personal (age, gender, qualification) [12,13] and contextual factors (e.g., educational targets, type of teaching) [14,15], indicating their role as predictors of competence development. Accordingly, competence assessment should be complemented by examining these factors to support digital sovereignty [16].

While digital competences have primarily been examined in educational contexts, they have increasingly gained attention in health sciences. This includes empirical studies and systematic reviews on digital competences, for example, in the context of nurses and healthcare professionals [17], as well as in disciplines such as rehabilitation [18]. However, these approaches are only partially transferable to the context of obesity therapy, which is characterized by a high degree of interdisciplinarity, involving professionals such as nutritionists, physicians, exercise therapists, and psychologists, each requiring distinct domains of digital competence. Within this setting, exercise therapists represent a central professional group, as exercise accounts for a substantial proportion of treatment and uniquely integrates theoretical and practical components. Despite this, empirical evidence on the digital competences of obesity exercise therapists remains scarce. To address this gap, the DigCompThExO framework was recently developed based on DigCompEdu and validated for obesity therapy [19]. The instrument comprises four dimensions (‘Selection Criteria’, ‘Teaching Strategies’, ‘Learning Support’, ‘Media Reflection’) and captures relevant personal (age, gender, professional qualification) and contextual determinants, including digital obesity exercise therapy (DOET) types and therapeutic targets derived from the model of physical activity-related health competences [20,21].

Despite its conceptual advancements, DigCompThExO and its personal and contextual predictors have not yet been evaluated in therapeutic practice [22]. This is critical: without such evidence, qualification strategies remain unguided, limiting digital sovereignty and the effective digital transformation of exercise therapy [23]. Consequently, this is the first study to apply DigCompThExO among digital obesity exercise therapists, providing an evidence-based foundation for targeted training and continuing education. Accordingly, the present study pursued the following research questions:

(1) How are digital competences distributed among exercise therapists in DOET?
(2) Which personal and contextual factors predict digital competences in DOET?

## 2. Materials and Methods

To answer these research questions, a structured methodological approach is required, such as the use of checklists. The present study is reported in accordance with established reporting guidelines to ensure transparency, quality, and completeness [24].

Study Design: This cross-sectional observational study was designed to assess self-perceived digital competences among obesity exercise therapists. Quantitative data collection was non-experimental and ex post facto in nature, aiming to distinguish itself from the predominantly qualitative competence studies available [25].

Setting: Data were collected between June 2023 and December 2024 using the DigCompThExO questionnaire implemented on the online platform LimeSurvey (LimeSurvey GmbH, Hamburg, Germany). Participants were recruited through professional associations, institutional mailing lists, rehabilitation centers offering digital aftercare, and continuing education networks across German-speaking countries. To ensure confidentiality, participation was fully anonymous. Given the cross-sectional design, no follow-up was conducted [26].

Participants: Eligible participants were German-speaking exercise therapists with current or prior involvement in exercise-based obesity therapy. Inclusion required engagement in the planning and delivery of therapeutic interventions, as well as at least one year of professional experience in exercise therapy. Electronic informed consent was obtained prior to participation, and a non-probabilistic purposive sampling approach was used.

Variables: The primary variables of interest were therapists’ self-assessed digital competences, measured with the DigCompThExO questionnaire, which captures four competence dimensions: ‘Selection Criteria’ (SC), ‘Teaching Strategies’ (TS), ‘Learning Support’ (LS) and ‘Media Reflection’ (MR) [19]. Potential predictors and confounders included personal variables (age, gender, qualification) and contextual factors (type of therapy and therapeutic targets), defined according to physical activity-related health competence [27].

Data sources/measurement: DigCompThExO operationalizes competence through 14 items across the four dimensions: SC evaluates strategies for selecting software (SC1), hardware (SC2), and data protection (SC3). TS assesses digital activation (TS1), the demonstration of educational DOET content (TS2), the delivery of practical DOET content (TS3), and the provision of feedback (TS4). LS captures the digital planning (LS1), documentation (LS2), and monitoring of DOET-related behavior (LS3). MR measures the reflective consideration of the purpose (MR1), mode (MR2), timing (MR3), and target group (MR4) of media use prior to therapy. Respondents rated their perceived competence on a six-point Likert scale. Psychometric validation confirmed factorial structure and adequate internal consistency [21]. Personal and contextual data were obtained via standardized self-report. Personal factors (n = 3) included gender, age, and the highest professional qualification. Contextual factors (n = 10) comprised the type of therapy (counseling, practical, and theoretical) and the therapeutic targets (motor abilities, motor skills, knowledge of effects, procedural knowledge, body awareness, self-efficacy, and motivation).

Study size: An a priori power analysis for multiple regression with 13 predictors (α = 0.05; power = 0.80) indicated that a minimum sample size of n = 131 would be required to detect medium effects (f^2^ = 0.15) according to Cohen’s conventional benchmarks for multiple regression, as no prior empirical estimates for the expected effect size in this context were available [28].

Quantitative variables: All competence items were treated as continuous [29,30], and an overall competence score was computed as the mean of the four dimensions. Descriptive statistics (mean, median, SD, IQR) were computed for overall and item-level distributions. Personal variables were numerically coded as follows: age was included as a continuous variable; gender and professional qualification were coded numerically, with qualification dichotomized into university degree (yes/no). Contextual factors were also assessed using a 6-point Likert scale (1 = does not apply; 6 = fully applies). Although ordinal, single-item Likert variables with five or more response categories are often treated as approximately continuous in regression analyses when sufficient variability is present. However, this approach assumes equal intervals between response categories and may be less robust than scale-based measures [27,31]. Alternative ordinal modeling approaches (e.g., ordinal logistic regression) were considered but deemed less suitable due to additional model assumptions (e.g., proportional odds), increased complexity, and reduced interpretability in multivariable contexts. Therapeutic target items, grounded in a shared theoretical framework and exhibiting sufficient variance, were therefore treated as continuous variables. Items reflecting modes of therapeutic implementation were included as single-item predictors, as they represent distinct components of the therapeutic setting, consistent with previous research [32,33]. This approach preserved information content and facilitated interpretation in multivariable analyses.

Statistical methods: Statistical analyses were conducted using R (version 4.5.1; R Foundation for Statistical Computing, Vienna, Austria). Descriptive statistics summarized central tendency and dispersion. Multiple linear regression models were estimated to examine the associations of personal and contextual factors with overall digital competence. Model assumptions were assessed using standard diagnostic procedures, including the inspection of residual distributions, linearity, homoscedasticity, multicollinearity, and influential cases [34]. Statistical significance was set at α = 0.05. Robust (HC3) standard errors were applied to account for potential heteroscedasticity [35]. Categorical predictors were dummy-coded with predefined reference categories. As this study was exploratory, interpretation focused on effect sizes and 95% confidence intervals, and *p*-values were adjusted using the Benjamini–Hochberg false discovery rate [36]. Sensitivity analyses were conducted to assess the robustness of the findings [37]. No subgroup or interaction analyses were performed.

Ethical considerations: This study was conducted in accordance with the Declaration of Helsinki and the institutional standards at Leipzig University. According to common institutional practice, studies involving fully anonymous, non-interventional surveys with adult participants and no foreseeable risk do not require formal ethics committee approval. A self-assessment was conducted by the authors to confirm that this study met these criteria. Participation was voluntary and anonymous, and informed consent was obtained from all participants prior to data collection.

## 3. Results

Descriptive statistics are presented first, followed by multiple regression analyses examining the personal and contextual predictors of digital competence.

Participants: In total, 246 exercise therapists responded to the online survey. After the exclusion of incomplete questionnaires (n = 43), the final sample comprised 203 participants for descriptive analyses. For the subsequent multivariate analyses, the sample was reduced to 202 included responses, as one participant identifying as non-binary had to be excluded due to insufficient group size for forming a distinct category within the regression models; accordingly, gender was treated as a binary variable (female, male) [38]. Due to the insufficient group size (n = 1), alternative analytical strategies were not feasible.

Study size: In light of the a priori estimated minimum sample size of n = 131, the final sample of 203 participants exceeded this threshold, ensuring sufficient statistical power to detect at least medium-sized effects [28].

Descriptive data: Data on 203 exercise therapists were included in the analyses (mean age = 33.3 ± 5.9 years; 52.2% male, 47.3% female, 0.5% diverse). Participants had an average of 7.1 ± 5.7 years of professional experience. Most were based in Germany (88.7%), with smaller proportions from Austria (8.4%) and other European countries (≤1% each). Nearly nine in ten participants (88.6%) held a university degree (38.9% Bachelor’s, 49.8% Master’s), while 6.4% were physiotherapists and 4.9% licensed sports trainers. Digital tools were the most frequently applied in practical (M = 5.44 ± 1.09) and theoretical therapy (M = 5.38 ± 1.02) and less often in counseling (M = 4.38 ± 1.22). Across therapeutic targets, media were primarily used to foster procedural knowledge (M = 5.38 ± 1.07), motor abilities (M = 5.35 ± 1.09), and knowledge of effects (M = 5.21 ± 1.04), while comparatively less emphasis was placed on motivation (M = 4.69 ± 1.13), motor skills (M = 4.66 ± 1.21), body awareness (M = 4.24 ± 1.11), and self-efficacy (M = 4.18 ± 1.19).

Outcome data: Descriptive outcome data are presented in Table 1. The overall competence score of the 203 participants averaged 4.73 ± 0.90 with a median of 5.04 and an interquartile range (IQR) of 0.67, indicating moderate variability. Dimension-level means ranged from 4.5 for SC to 5.0 for TS. At the item level, values spanned 3.7 (SC3, MR1) to 5.4 (TS2, TS3, MR4), consistent with the corresponding medians and IQRs, suggesting that the results were not affected by extreme values.

Main results: Multiple linear regression analyses were conducted for 202 participants. Model diagnostics indicated heteroscedasticity; therefore, HC3 robust standard errors were applied [39]. Influence diagnostics (Cook’s D ≤ 0.64) identified 20 potentially influential cases; excluding them did not alter the results, confirming the robustness of the findings (see Other analyses) [40]. The model explained a substantial proportion of variance in overall digital competence (R^2^ = 0.801, adjusted R^2^ = 0.79; F(13, 188) = 60.61, *p* < 0.0001) [41]. Using HC3-adjusted standard errors, significant positive predictors were the therapeutic targets ‘motor abilities’ (B = 0.18, *p* = 0.009) and ‘body awareness’ (B = 0.18, *p* < 0.001), as well as ‘theoretical therapy’ (B = 0.20, *p* = 0.026). All other predictors, including sociodemographic variables, were non-significant. The complete regression results are shown in Table 2 († *p* < 0.10 (indicating a trend), * *p* < 0.05, ** *p* < 0.001).

After false discovery rate (FDR) correction, ‘body awareness’ remained the only significant predictor of overall competence (B = 0.18, 95% CI [0.08, 0.27], p_FDR = 0.003) [42]. Two further predictors, ‘motor abilities’ and ‘theoretical therapy’, which were significant in the unadjusted model (*p* = 0.009 and *p* = 0.26, respectively), lost significance after correction (p_FDR = 0.066 and 0.121). All other predictors were non-significant.

Other analyses: Sensitivity analyses excluding influential cases identified by Cook’s distance confirmed the robustness of the main findings. The core predictors (‘body awareness’, ‘motor abilities’, and ‘theoretical therapy’) remained significantly associated with overall digital competence, while sociodemographic variables remained non-significant. Some secondary predictors showed changes in significance, indicating sensitivity to influential observations.

## 4. Discussion

This study provides an overview of the digital competences relevant for planning and implementing digital obesity exercise therapy. Therapists showed the highest competence in TS and the lowest in SC, indicating strengths in digitally delivering exercise content but weaknesses in data protection and reflective media use. Competence levels were primarily associated with contextual rather than personal factors, with digitally fostering ‘body awareness’ emerging as the only significant predictor after FDR correction.

### 4.1. Profiling Digital Competence of DOET Therapists

Overall, therapists demonstrated high competence levels, with variation across dimensions. Competence was high in ‘Teaching Strategies’, underscoring therapists’ confidence in using digital media to explain and demonstrate exercise-based therapeutic content. This likely reflects the interactive nature of exercise therapy, where digital competence is primarily expressed through patient-centered engagement and the ability to facilitate shared therapeutic processes [43]. Yet contrasting evidence reveals that physiotherapy educators often lack the didactic strategies required to embed technology meaningfully into instruction [44] and that current training frameworks prioritize administrative over pedagogical digital competences [45]. Thus, although therapists may use digital media extensively, this does not guarantee didactic mastery within their professional domain. This indicates challenges in the domain-specific application of digital media and underscores the urgent need for systematic quality assurance in digital therapy.

‘Learning Support’ ranked second, likely reflecting therapists’ familiarity with digital tracking through the everyday use of wearables or health apps, which may transfer readily into therapy [46,47]. Yet this apparent strength may be misleading: many consumer devices provide inaccurate data (e.g., for energy expenditure), potentially promoting misguided feedback [48]. The extent to which therapists recognize and communicate these risks remains unexplored. Moreover, digital learning support demands self-regulation, intrinsic motivation and media acceptance of patients [49,50]. Without consistent therapeutic support, dropout risk rises, underscoring the need for well-integrated digital tools within guided therapy [51]. Future studies should explore how learning support is delivered and how to balance professional synchronous guidance with autonomous asynchronous digital learning to sustain engagement [52].

Low scores in ‘Selection Criteria’ reveal persistent challenges in digitizing haptic, interaction-based exercise therapy. Unlike educational settings with frameworks for non-haptic instruction [53,54], exercise professionals lack comparable guidance, fostering uncertainty and fragmented tool use [55]. Consequently, many master one digital tool but struggle to transfer competences across formats, as growing media diversity amplifies cognitive load rather than competence [44,56]. Also, structural barriers, such as institutional mandates or limited funding, often confine therapists to a single, often user-unfriendly platform [57,58]. Such constraints limit media-reflective competence, as therapists cannot evaluate the suitability of alternative tools, and simultaneously reduce opportunities for context-specific tool adaptation and application, despite its critical role in supporting patient adherence [59,60]. This gap undermines the need to strengthen therapists’ selection competences and integrate them into digital design processes [61].

‘Media Reflection’ revealed ambivalence. While strong reflection on patient groups reflects therapists’ habitual focus on individual needs (a transferable core of clinical work [62]), limited reflection on media purpose indicates pragmatic reliance on mandated tools rather than critical, value-driven use [63]. Although media are recognized as beneficial for monitoring and self-management [64,65,66,67], ongoing concerns about quality and workload lead many therapists to view technology as administrative rather than therapeutic [68,69,70]. In this context, therapists strongly identify with their sport science education as core competence, while the lack of informatic training is perceived as a barrier to developing digital competencies [21]. This limited acceptance weakens motivation and impedes the growth of self-reflective attitude, emphasizing the need to foster more positive attitudes toward media use [71,72]. The lack of reflection frameworks compounds this issue, whereas evidence shows that peer observation and guided feedback foster self-evaluation and innovation [73,74]; embedding such approaches in exercise therapy could transform digital tools from compliance instruments into drivers of engagement and therapeutic progress.

### 4.2. Personal Predictors of DOET Therapists’ Digital Competence

Consistent with prior research, a non-significant negative trend was observed, suggesting that higher age might be associated with slightly lower digital competence [75,76]. Although age is often linked to lower competence [77], such effects appear largely indirect: older professionals experience higher digital stress [78] and have had less exposure to structured digital training now embedded in modern education [11,76]. Kerzic et al. [79] further suggest that age affects instructional media use but not overall engagement, indicating domain-specific rather than general age effects. In this study, the absence of age effects likely reflects the sample’s youth and digital socialization [80]. This bias may stem from recruitment, with older therapists being underrepresented in online surveys and less involved in digital therapy than younger ‘technology champions’ [81]. Given limited evidence on older therapists, future research should examine age-related competence to better include this group in digital transformation and sustain workforce innovation.

No significant gender effects were found. Although earlier studies identified a self-concept gap with men overestimating and women underestimating their abilities [82,83], this study supports evidence of a growing convergence in digital competence between men and women as digitalization progresses [79,84]. Beyond binary gender perspectives, the exclusion of diverse participants due to low case numbers underscores a broader research gap in terms of digital competences among gender-diverse therapists. Evidence suggests that non-binary persons use digital environments more frequently to compensate for limited social support and show higher competence in digital content creation [85,86]. These populations remain underexplored yet offer valuable insights [87]. Future research should therefore address the ethical consideration of gender inclusivity by adopting study designs that enable the adequate representation and analysis of diverse gender identities.

Although evidence suggests that higher academic qualifications are associated with higher digital competence [76], no such association was found in this study. This divergence likely arises because postgraduate education strengthens theoretical rather than practical digital competences [88,89]. In contrast, movement-related disciplines rely more on experiential learning, where the hands-on use of digital tools builds confidence and applied competence [90]. These findings imply that practical engagement, rather than theoretical instruction, may be the key driver of digital competence in exercise therapy, highlighting the importance of empirically grounded, practice-oriented training approaches.

### 4.3. Contextual Predictors of DOET Therapists’ Digital Competence

The frequent usage of media in theoretical therapy predicted higher digital competence before FDR correction. While DOET integrates theoretical and practical elements [91], clinical routines remain dominated by motor instruction, with education serving a complementary role [92,93]. Higher competence in educational formats likely reflects the greater standardization of tools (mainly videoconferencing platforms such as Zoom), facilitating intuitive use and reducing cognitive load [94,95,96]. Conversely, practical applications involve heterogeneous, poorly standardized tools [97,98], hindering transferable digital proficiency. As theoretical content adapts more readily to digital delivery, a competence–action gap persists in virtual motor instruction [99]. Though significance diminished after FDR correction, the trend highlights the need for greater standardization in practical digital therapy to strengthen applied competence and ensure consistent DOET. Interestingly, higher levels of knowledge of effects were associated with a slight, non-significant decrease in digital competence scores. This may reflect a more critical, evidence-based perspective toward the effects of digital interventions, particularly where empirical support and standardization remain limited [100]. Accordingly, higher effect knowledge may reflect a more differentiated appraisal of digitally delivered exercise therapy, warranting further investigation.

The therapeutic target of digitally fostering body awareness was the only variable significantly associated with digital competence in this study. This may indicate a potentially relevant, yet complex, role in digital exercise therapy. However, this association should be interpreted with caution given the comparatively large explained variance and the study design and also because fostering body awareness ranked second to last among the seven therapeutic goals pursued through digital media. One possible explanation may be that body awareness is addressed less frequently in practice, for example, due to concerns about patients’ emotional responses, with therapists instead focusing on physical function [101], an approach that improves body composition but not awareness. In digital settings, these challenges are intensified: empathy is harder to convey without nonverbal cues, and constant digital self-viewing may amplify self-criticism [102,103], underscoring the need for integrated emotional, communicative, and technical training in therapist education [77]. Future research may examine how body awareness can be addressed in digitally mediated contexts to support therapist confidence and patient engagement despite physical distance.

### 4.4. Limitations

This study has several limitations.

First, the cross-sectional design precludes causal inference [41]. In addition, the use of single-time-point self-report measures for both predictors and outcomes may increase the risk of common method bias, potentially inflating observed associations.

Second, participants were recruited using non-probabilistic purposive sampling, which may limit the representativeness of the sample and introduce self-selection bias, potentially leading to the overrepresentation of younger and more digitally competent therapists. The focus on German-speaking exercise therapists further limits the transferability of the findings to other professional groups, cultural contexts and other healthcare systems with different training structures, treatment approaches and digital infrastructures, particularly in less developed settings. In addition, although a minimum level of professional experience was required, variations in experience duration were not systematically analyzed and may have influenced the findings. Future research should therefore consider stratifying participants by experience level to allow for more differentiated analyses.

Third, self-reported data may be affected by social desirability, potentially leading to overestimated and less variable competence scores [104]. As digital competence was assessed exclusively via self-perception, the results may reflect perceived rather than actual competence and introduce response biases, particularly among digitally engaged participants. The absence of objective competence assessments also limits validation, and measurement objectivity may have been influenced by contextual factors (e.g., stress [105]).

Fourth, while the sample size was sufficient to detect moderate effects within the binary gender groups, smaller effects may have remained undetected [106]. However, the gender-diverse participant could not be included in the analyses due to insufficient sample size, highlighting ethical considerations regarding gender inclusivity in research.

Fifth, the overall competence score included items conceptually related to some predictors (e.g., ‘body awareness’), reflecting a degree of conceptual proximity between variables and, to some extent, a potential risk of circularity. This may partly account for the comparatively large proportion of explained variance (R^2^ = 80.1%) and warrants cautious interpretation. The observed R^2^ may further reflect shared measurement characteristics, sample characteristics (e.g., a potentially higher digital affinity among participants) and aspects of model specification, such as the inclusion of multiple theoretically related predictors, as well as potential overfitting, despite acceptable diagnostic results. Furthermore, the interpretation of body awareness as a sole predictor should be treated with caution, as variations in professional training and specialization among therapists may have influenced the emphasis on this therapeutic target and the reported levels of digital competence.

Finally, contextual factors were assessed using single-item measures, and not all potentially relevant confounders (e.g., heterogeneous media use) were captured [107].

### 4.5. Implications

These findings provide initial indications for conceptual implications related to the development of digital competences and for policy-related considerations regarding their implementation in therapeutic practice.

From a conceptual perspective, the findings suggest the potential relevance of didactic strategies for digital media use across both synchronous (e.g., videoconferencing) and asynchronous (e.g., self-guided learning or tracking) settings to enhance flexibility in therapeutic delivery. Literacy in data protection emerges as an important aspect, as the ethical management of patient information remains a fundamental yet underdeveloped competence. Furthermore, a deeper understanding of digital tool diversity may support context-sensitive choices and build confidence in their use. Lastly, the findings indicate that attention to body awareness could play a role in digital exercise therapy. Future work may therefore explore how body awareness can be addressed in digitally mediated contexts. Taken together, these considerations point to potential areas for the conceptual development of digital competences.

Beyond these conceptual implications, several practical and policy-related priorities remain. The identified digital competence profiles provide initial insights into potential gaps in therapists’ competences. Here, future studies should further examine digital competences and their predictors using separate measurement approaches to reduce the risk of common method bias. Such findings may then inform the development of targeted training approaches for professional education and continuing training. Future research should evaluate the effectiveness of such training approaches to examine whether they lead to measurable changes in therapists’ competences. In addition, further conceptual and operational work is needed to refine digital sovereignty as a curricular target and to better align digital competence development with autonomous digital practice. If supported by empirical evidence, such training developments could contribute to facilitating the implementation of digital obesity therapy. Extending competence assessments to other populations, including different age groups and professional disciplines within obesity care, may further advance research in this field.

## 5. Conclusions

This study provides initial empirical insight into the digital competence profile of DOET therapists, revealing challenges in applying data protection and reflecting on the purpose of media use. Notably, the therapeutic target of digitally fostering ‘body awareness’ emerged as the only variable that remained significantly associated with digital competence after correction. These findings provide initial indications for the development of targeted training approaches to strengthen therapists’ technological, communicative, and reflective competences. However, these implications should be interpreted in light of the conceptual proximity between variables and this study’s measurement approach. In this context, advancing digital competences provides a basis for fostering digital sovereignty among exercise therapists.

## Figures and Tables

**Table 1:** Descriptive statistics of competences for planning and implementing digital obesity exercise therapy, assessed with DigCompThExO.

Dimension	Item	M	SD	Md (Q1–Q3)	IQR
Selection Criteria (SC)	Software Selection	4.9	1.2	5.0 (4.0–6.0)	1.0
	Hardware Selection	4.8	1.2	5.0 (4.0–6.0)	2.0
	Data Protection Strategies	3.7	1.1	4.0 (3.0–4.0)	1.0
	SC (total)	4.5	1.0	4.7 (4.3–5.3)	1.0
Teaching Strategy (TS)	Digital Activation	4.9	1.2	5.0 (4.0–6.0)	2.0
	Demonstrating Practice	5.4	1.1	6.0 (5.0–6.0)	1.0
	Explaining Theory	5.4	1.1	6.0 (5.0–6.0)	1.0
	Providing Feedback	4.3	1.2	5.0 (4.0–5.0)	1.0
	TS (total)	5.0	1.0	5.3 (4.8–5.5)	0.8
Learning Support (LS)	Media for Behavior Planning	4.9	1.3	5.0 (4.0–6.0)	2.0
	Media for Behavior Documentation	5.1	1.3	6.0 (5.0–6.0)	1.0
	Media for Behavior Monitoring	4.6	1.3	5.0 (4.0–5.0)	1.0
	LS (total)	4.8	1.2	5.3 (4.7–5.7)	1.0
Media Reflection (MR)	Reflection on Purpose	3.7	1.0	4.0 (3.0–4.0)	1.0
	Reflection on Method	4.8	1.2	5.0 (5.0–6.0)	1.0
	Reflection on Timing	4.6	1.1	5.0 (4.0–5.0)	1.0
	Reflection on Target Group	5.4	1.2	6.0 (5.0–6.0)	1.0
	MR (total)	4.6	0.9	5.0 (4.5–5.0)	0.5

Legend: M = mean; SD = standard deviation; Md = median; Q1 = first quartile; Q3 = third quartile; IQR = interquartile range.

**Table 2:** Multiple regression on exercise therapists’ competence.

Variable		B	SE	t	95% CI		*p*
					LL	UL	
Gender ^a^		0.04	0.07	0.62	−0.09	0.17	0.536
Age		−0.001	0.01	−0.23	−0.01	0.01	0.819
Qualification ^b^		0.02	0.11	0.16	−0.20	0.23	0.870
Type of therapy	Practical therapy	0.06	0.07	0.85	−0.08	0.21	0.397
	Theoretical therapy	0.20	0.09	2.25	0.02	0.37	0.026 *
	Counseling	0.08	0.06	1.50	−0.03	0.19	0.135
Therapeutic target	Motor abilities	0.18	0.07	2.62	0.05	0.32	0.009 *
	Motor skills	0.09	0.05	1.89	−0.004	0.19	0.060 †
	Knowledge of effects	−0.11	0.08	−1.42	−0.26	0.04	0.158
	Procedure knowledge	0.01	0.06	0.21	−0.11	0.13	0.834
	Body awareness	0.18	0.05	3.79	0.08	0.27	>0.001 **
	Self-efficacy	0.06	0.05	1.20	−0.04	0.16	0.230
	Motivation	0.12	0.08	1.52	−0.04	0.28	0.129

Legend: B = unstandardized regression coefficient; SE = robust standard error (HC3); t = test statistic; CI = confidence interval; LL = lower limit; UL = upper limit; *p* = *p*-value; † *p* < 0.10 (trend), * *p* < 0.05, ** *p* < 0.001. Reference categories: ^a^ 0 = female, 1 = male; ^b^ 0 = no university degree, 1 = university degree.

## Data Availability

The datasets generated during and/or analyzed during the current study are not publicly available due to data protection and ongoing analysis but are available from the corresponding author on reasonable request.

## Abbreviations

The following abbreviations are used in this manuscript:

B	Unstandardized regression coefficient
CI	Confidence interval
Df	Degrees of freedom
DigCompEdu	Digital Competence Framework for Educators
DigCompThExO	Digital Competence Framework for Exercise Therapists in obesity treatment
DOET	Digital obesity exercise therapy
F	F-statistic (model fit statistic)
F^2^	Cohen’s f^2^ (effect size)
FDR	False discovery rate
HC3	Heteroskedasticity-consistent standard error estimator type 3
IQR	Interquartile range
LL	Lower limit (of confidence interval)
LS	Learning support, dimension of DigCompThExO
M	Mean
Md	Median
MR	Media reflection, dimension of DigCompThExO
n	Sample size
P	*p*-value
P_FDR	FDR-adjusted *p*-value
Q1	First quartile
Q3	Third quartile
Q-Q Plot	Quantile–quantile plot
R^2^	Coefficient of determination
SC	Selection criteria, dimension of DigCompThExO
SD	Standard deviation
SE	Robust standard error
t	*t*-statistic (for regression coefficients)
TS	Teaching strategy, dimension of DigCompThExO
UL	Upper limit (of confidence interval)
